# Subregional statistical shape modelling identifies lesser trochanter size as a possible risk factor for radiographic hip osteoarthritis, a cross-sectional analysis from the Osteoporotic Fractures in Men Study

**DOI:** 10.1016/j.joca.2020.04.011

**Published:** 2020-08

**Authors:** B.G. Faber, T.L. Bredbenner, D. Baird, J. Gregory, F. Saunders, C.V. Giuraniuc, R.M. Aspden, N.E. Lane, E. Orwoll, J.H. Tobias

**Affiliations:** † Medical Research Council Clinical Research Fellow, Musculoskeletal Research Unit, University of Bristol, Bristol, UK; ‡ Mechanical and Aerospace Engineering, University of Colorado Colorado Springs, Colorado, USA; § Integrative Epidemiology Unit, University of Bristol, Bristol, UK; ‖ Centre for Arthritis and Musculoskeletal Health, University of Aberdeen, Aberdeen, UK; ¶ Center for Musculoskeletal Health, U.C. Davis School of Medicine, Sacramento, CA 95817, USA; # Bone and Mineral Unit, Oregon Health and Sciences University, Portland, OR, USA; †† Musculoskeletal Research Unit, University of Bristol, Bristol, UK

**Keywords:** Osteoarthritis, Hip shape, Statistical shape modelling, Epidemiology

## Abstract

**Objective:**

Statistical shape modelling (SSM) of hip dual-energy X-ray absorptiometry (DXA) scans has identified relationships between hip shape and radiographic hip OA (rHOA). We aimed to further elucidate shape characteristics related to rHOA by focusing on subregions identified from whole-hip shape models.

**Method:**

SSM was applied to hip DXAs obtained in the Osteoporotic Fractures in Men Study. Whole-hip shape modes (HSMs) associated with rHOA were combined to form a composite at-risk-shape. Subsequently, subregional HSMs (cam-type and lesser trochanter modes) were built, and associations with rHOA were examined by logistic regression. Subregional HSMs were further characterised, by examining associations with 3D-HSMs derived from concurrent hip CT scans.

**Results:**

4,098 participants were identified with hip DXAs and radiographs. Composite shapes from whole-hip HSMs revealed that lesser trochanter size and cam-type femoral head are related to rHOA. From sub-regional models, lesser trochanter mode (LTM)1 [OR 0.74; 95%CI 0.63.0.87] and cam-type mode (CTM)3 [OR 1.27; 1.13.1.42] were associated with rHOA, associations being similar to those for whole hip HSMs. 515 MrOS participants had hip DXAs and 3D-HSMs derived from hip CT scans. LTM1 was associated with 3D-HSMs that also represented a larger lesser trochanter [3D-HSM7 (beta (*β*)-0.23;-0.33,-0.14) and 3D-HSM9 (β0.36; 0.27.0.45)], and CTM3 with 3D-HSMs describing cam morphology [3D-HSM3 (β-0.16;-0.25,-0.07) and 3D-HSM6 (*β* 0.19; 0.10.0.28)].

**Conclusion:**

Subregional SSM of hip DXA scans suggested larger lesser trochanter and cam morphology underlie associations between overall hip shape and rHOA. 3D hip modelling suggests our subregional SSMs represent true anatomical variations in hip shape, warranting further investigation.

## Introduction

Osteoarthritis (OA) affects 250 million people worldwide with the hip being the third most commonly effected joint^1^. Hip OA causes significant pain and morbidity^2^ leading to 80,000 hip replacements each year in England and Wales alone^3^. For the purpose of epidemiological studies, hip OA can be defined either clinically or radiologically. Although these two measures are known to be discordant^4^, nevertheless, radiological measures of OA have a degree of clinical relevance given their relationship with risk of total hip replacement^5^. Currently, we are poor at predicting the onset and preventing progression of hip OA which underlines the importance of understanding risk factors for it so that new interventions can be developed to decrease the impact of this disease.

Hip shape variations, in the form of developmental dysplasia of the hip (DDH)^6^, femoro-acetabular impingement (FAI) syndrome comprising cam and pincer morphologies measured geometrically^7^ and similar morphologies measured via statistical shape modelling (SSM), have strong associations with hip OA^8^^,^^9^. Better knowledge of these shape variations and their origins could potentially offer new pathways to prediction^10^^,^^11^ and prevention of hip OA, the later based on interventions that mediate the effects of hip shape^12^.

SSM is a modelling approach that conducts principal component analysis (PCA) on points placed around objects of interest in images to create a set of statistically derived shape variations termed modes. SSM was first used on hip radiographs to quantify hip shape before being used on hip dual-energy X-ray absorptiometry (DXA) images, with both applications showing associations between hip shape modes (HSMs) and the presence of hip OA^8^^,^^9^^,^^13^. The OA-associated HSMs from previous studies have shown various shape variations consistent with a cam morphology, pincer morphology, retroverted acetabulum, larger greater trochanter and larger lesser trochanter^8^^,^^9^^,^^14^^,^^15^. Each mode describes features that vary in a coordinated fashion and each is independently associated with OA, so one mode can incorporate several shape features. For example, in previous work MrOS HSM1 described the sizes of the greater and lesser trochanters and pincer-type morphology, making it hard to know exactly which shape variations are important relative to the pathomechanics of radiographic hip OA (rHOA)^9^. Subregional models of joint shape which concentrate on one distinct area of anatomy, such as joint space or the lateral femoral head, are more tractable and sensitive. This was demonstrated recently when exploring the genetic influences on hip shape with the gene for disruptor of telomeric silencing 1-like (DOT1L) protein. Investigators reported that this polymorphism was associated with a reduced superior joint space seen on subregional modelling, and this result was not evident when looking at whole hip shape models^16^.

SSM from either DXA scans or radiographs provides a 2-dimensional (2D) hip shape model but SSM can also be applied to computed tomography (CT) scans to form 3-dimensional (3D) hip shape models. 3D hip shape measured by SSM is increasingly viable as computational power improves but is restricted to small cohorts due to the increased cost and radiation exposure involved in acquiring the CT scans. Recently, 3D SSM was used to show the difference in femoral shape between DDH and controls^17^ and, previously, 3D SSM combined with density measures (statistical shape and density modelling) was used to show associations between hip shape and density and the risk of hip fracture^18^. Cohorts with both 2D and 3D hip shape data provide an opportunity to explore whether 2D hip shape models accurately reflect the underlying shape of the hip or whether they are affected by inadequate positioning during image acquisition.

This study's aim was to extend our previous cross-sectional analysis of DXA-derived hip shape in the Osteoporotic Fractures in Men (MrOS) Study^9^ by combining whole-hip HSMs associated with prevalent rHOA to visualise a composite at-risk-shape for prevalent rHOA, providing a basis for developing subregional models which focus on key anatomical areas of interest. We then aimed to examine how HSMs generated from these subregional models relate to rHOA, and to further characterise the anatomical features they represent based on relationships with 3D-HSMs derived from concurrent hip CT scans.

## Methods

### Study participants

The MrOS study is a prospective cohort of men recruited between 2000 and 2002 at six centres around the United States (Birmingham, Alabama; Minneapolis, Minnesota; Palo Alto, California; the Monongahela Valley near Pittsburgh, Pennsylvania; Portland, Oregon; and San Diego, California). Eligibility requirements were: male sex, age ≥65 years old, ambulatory and without bilateral hip replacements. A full description has been previously published^19^^,^^20^ and a comprehensive data catalogue is available online (http://mrosdata.sfcc-cpmc.net). We used hip shape data derived from DXA scans performed at the baseline visit (Hologic QDR 4500 machine, Waltham, MA). For each DXA scan, the individual was positioned with their hip at 25° internal rotation. Pelvic radiographs assessing for rHOA were obtained as part of a second visit conducted from March 2005 to May 2006, on average 4.6 years later. We analysed only those individuals with both a right hip DXA and right hip measurements from their pelvic X-ray, excluding those who had right hip replacements and incomplete covariates.

### Demographic characteristics

All demographic information used in this analysis was obtained at the baseline MrOS visit. The participant's age was taken as the age in years at their last birthday. Height was measured in centimetres with a Harpenden stadiometer (Holtain Ltd, Crynch, Wales) and was based on an average of two readings. Weight was measured to the nearest 0.1 kg using a standard balance–beam scale or digital scales using standard protocols. The race was a self-identified criterion with the participants selecting one of the following: White, African American, Asian, Hispanic or other.

### Statistical shape modelling

**Fig. 1:**
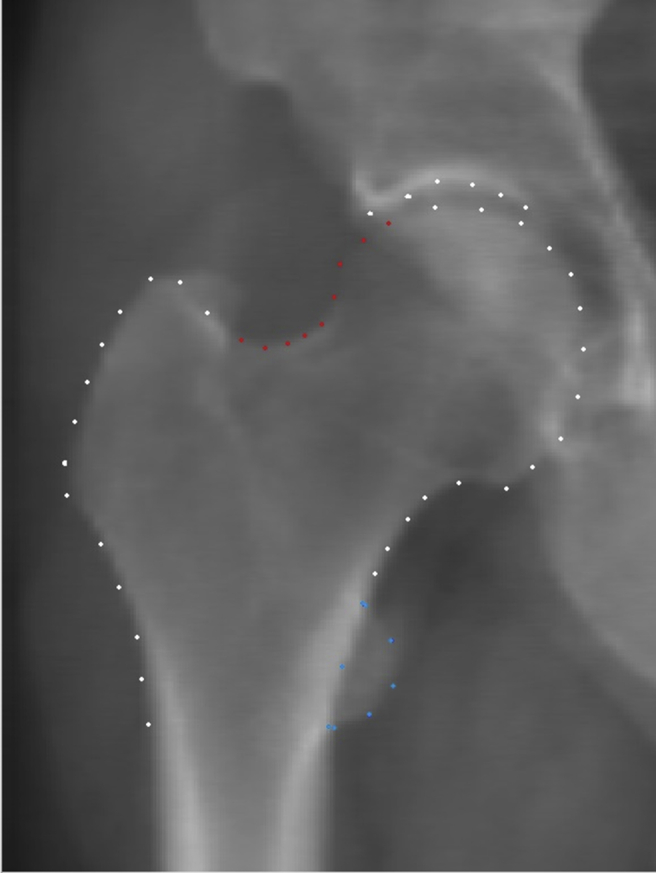
An example DXA image, This is a DXA image taken from the MrOS cohort. All the 53 points used for the SSM are marked on the image (combination of all white, red and blue points). Subregional models were composed of a smaller selection of points, the cam-type model comprising the red points and the lesser trochanter model comprising the blue points.

For our SSM we used the 53-point model developed by Baird *et al.* for their hip shape genome-wide association study^21^. Briefly, this was built from hip DXA scans from five cohorts (*n* = 19,379): the Osteoporotic Fractures in Men (MrOS) Study^19^^,^^22^, the Study of Osteoporotic Fractures (SOF)^23^, Framingham Osteoporosis Study (FOS)^24^, TwinsUK^25^^,^^26^, and the Avon Longitudinal Study of Parents and Children (ALSPAC; mothers’ first images taken)^27^^,^^28^. Shape software (custom, proprietary software for SSM, University of Aberdeen https://w3.abdn.ac.uk/clsm/shape/, freely available to all verified researchers (^14^,^29^)) placed 58 points automatically around the upper femur and adjacent acetabulum with a trained operator checking each image for correct point placement^9^. Five points were excluded following post processing, four on the femoral shaft below the lesser trochanter which varied greatly depending on how much femur was imaged and one point at the lateral aspect of the acetabulum designed to capture osteophytes that were not often visible, further details have previously been published^30^. This left 53 points with 12 anatomically guided key points and 41 equally spaced points (Fig. 1) for our SSM^30^. Procrustes analysis was performed to transform the points without deformation by scaling, rotation and translation so that they are aligned as closely as possible, this was followed by Principal Component Analysis (PCA)^9^. This whole process termed SSM produces linearly independent variations in hip shape (HSM)^14^^,^^31^ that are ranked in decreasing order of variance explained. Each mode was normalized to zero mean and unit standard deviation for the whole cohort so that each image (and therefore participant) is assigned a set of mode scores in units of standard deviations (SD). Further quality control was applied to images producing HSM scores above or below 4SDs with two operators checking image quality and point placement together. To reduce the multiple correction burden when making statistical comparisons, the top 10 HSMs (previously published^21^) were selected as they explained the majority of the total shape variance (>85%, a threshold we previously used^9^^,^^21^). Each excluded HSMs only described a small portion of variance in the sample (<1%).

To visualise a composite at-risk-shape, we combined HSMs associated with rHOA that met the significance threshold (Bonferroni-corrected *P*-value <0.005 to account for the 10 HSMs tested), weighting these according to their effect sizes (derived from the natural log of the odds ratio (OR) with their relationship with rHOA) and amplified these effects five times to allow clearer visualisation when plotting the shape differences. Mean hip shape specific to MrOS participants was subsequently generated from the five cohort SSM, based on previously reported mean HSM scores^21^.

The regions showing pronounced shape difference between the composite at-risk-shape and mean MrOS hip shape were selected for subregional modelling. Subregional models were built from subsets of points taken from the 53 point model which were felt to encompass the areas of interest (Fig. 1) following an approach that was previously reported^16^ and using the same point subset to model the lateral femoral head, and again limiting our testing to those modes that explained >85% total shape variance. These subregional mode scores are denoted by lesser trochanter mode (LTM) and cam-type mode (CTM) respectively and all the subregional modes analysed are displayed in the supplementary information (Supplementary Figs. 1 and 2).

### Radiographic measures of OA

At visit two, standing pelvic radiographs were recorded using a standardized protocol^32^. Each radiograph was read by a primary reader and scored, using a previously published atlas^33^, for features of rHOA namely osteophytes, joint space narrowing (JSN), subchondral sclerosis and cysts^32^. An aggregate of these scores led to a Croft Score for each hip^34^, with a score ≥2 (requiring the presence of osteophytes or JSN and another feature of rHOA – osteophytes, JSN, subchondral sclerosis, subchondral cysts) taken as the presence of rHOA^32^.

### 3-Dimensional hip shape

At baseline, quantitative CT scans were taken on 3,561 individuals. In previous work considering the prediction of fracture risk in the proximal femur, a case-cohort sampling design was used to select a subsample with a total of 518 men^18^. Briefly, baseline CT scans for these individuals were segmented to extract right femur data and triangulated surfaces were generated from the segmented CT data. Surface correspondence was optimized using an objective function based on minimum description length^35^^,^^36^ and 3D-HSMs were generated from a 3-dimensional (3D) statistical shape model describing variation in proximal femur geometry^18^. The top 20 3D-HSMs examined in our analysis are displayed using heat maps which represent geometric differences associated with one positive standard deviation away from the mean femur (Supplementary Fig. 3).

### Statistical analysis

Demographic statistics are presented as mean (SD) for continuous variables and counts (percentages) for categorical variables. We modelled HSMs, both whole and subregional, as exposures and rHOA (Croft score ≥2) as our initial analysis using logistic regression. After consideration of possible causal pathways using directed acyclic graphs, these models were additionally adjusted *a priori* for site of MrOS assessment, age, height, weight and race as recorded at visit one, because of either previously reported^1^^,^^37^^,^^38^ or plausible independent associations with both hip shape and rHOA. Effect sizes are expressed as odds ratios (OR) with 95% confidence intervals (CI). Multiplicity is a feature of SSM studies and to reduce this we limited the number of HSMs examined using a percentage variance threshold (previously mentioned) and used a Bonferroni corrected *P*-value, adjusted by the number of HSMs examined in each analysis. Top findings in our initial analysis exploring whole HSMs were reported using a Bonferroni-corrected *P*-value of 0.005 (alpha (α) = 0.05, *n* = 10 tests to account for the top 10 HSMs examined). In subsequent subregional analyses the same threshold was kept as we applied the same number of independent tests (2 subregional models examined 5 HSMs each). Our exploratory analysis examining the association between the 2 subregional modes of interest and the top 20 3D-HSMs, modelled using linear regression, used a Bonferroni-corrected *P*-value of 0.00125 (α=0.05, *n* = 40 tests, 2 subregional HSMs tested against 20 3D-HSMs). We estimated variance of 3D-HSMs explained by our 2D hip shape model using linear regression. All statistical analysis used Stata release 14 statistical software (StataCorp, College Station, TX, USA).

## Results

### Population characteristics

MrOS enrolled a total of 5,994 men at baseline. 4,098 individuals attended for visit 2, on average 4.6 years later, and had a right hip radiograph read for the presence of rHOA were included in this study. Participants were a mean of 72.8 years of age, 83.6 kg in weight, and 174.4 cm in height, giving a mean BMI of 27.5 kg/m^2^. Their self-reported race was 90.4% white, 3.3% Asian, 3.2% African American, 1.9% Hispanic and 1.3% Other. At visit 2, 7.0% had evidence of rHOA, based on Croft score ≥2. The subgroup of 515 participants with 3D-HSMs data were similar across these measures apart from having a lower prevalence of OA (5.4%) (Table I).

**Table I:** Demographics of the two samples used in the analysis

	2D hip shape sample	3D hip shape sample	
**Demographics**	Mean [Range]	Mean [Range]	
Age (years)	72.8 [64–93]	73.9 [65–92]	
Weight (kg)	83.6 [48.5–144.1]	83.2 [55.3–128.9]	
Height (cm)	174.4 [151.8–198.9]	174.4 [147.2–197.7]	
*Race*	Prevalence [%]	Prevalence [%]	
White	3,704 [90.4]	454 [88.2]	
African American	130 [3.2]	19 [3.7]	
Asian	136 [3.3]	16 [3.1]	
Hispanic	76 [1.9]	18 [3.5]	
Other	52 [1.3]	8 [1.6]	
*Site of investigation*			
Birmingham	674 [16.5]	77 [15.0]	
Minneapolis	742 [18.1]	84 [16.3]	
Palo Alto	615 [15.0]	96 [18.6]	
Pittsburgh	667 [16.3]	89 [17.3]	
Portland	671 [16.4]	88 [17.1]	
San Diego	729 [17.8]	81 [15.7]	
**Radiographic OA**			
Croft score <2	3,810 [93.0]	487 [94.6]	
Croft score ≥2	288 [7.0]	28 [5.4]	
**Total**	4,098	515	

### SSM characteristics

The first 10 HSMs in the whole-hip model accounted for 86.1% total variation in 2D hip shape. The first 5 LTMs accounted for 85.6% and the first 5 CTMs captured 85.7% of the total shape variation captured by their respective models (Supplementary Fig. 1&2). Our first 20 3D-HSMs explained 91.1% of 3D shape variance (Supplementary Fig. 3). The shape variance explained by each 2D whole, subregional and 3D modes are shown in the supplementary material (Supplementary Fig. 4). The variance in 3D-HSMs explained by our 2D whole hip shape model are given in the supplementary material (Supplementary Table 1).

### Relationship between hip shape modes and radiographic OA

**Fig. 2:**
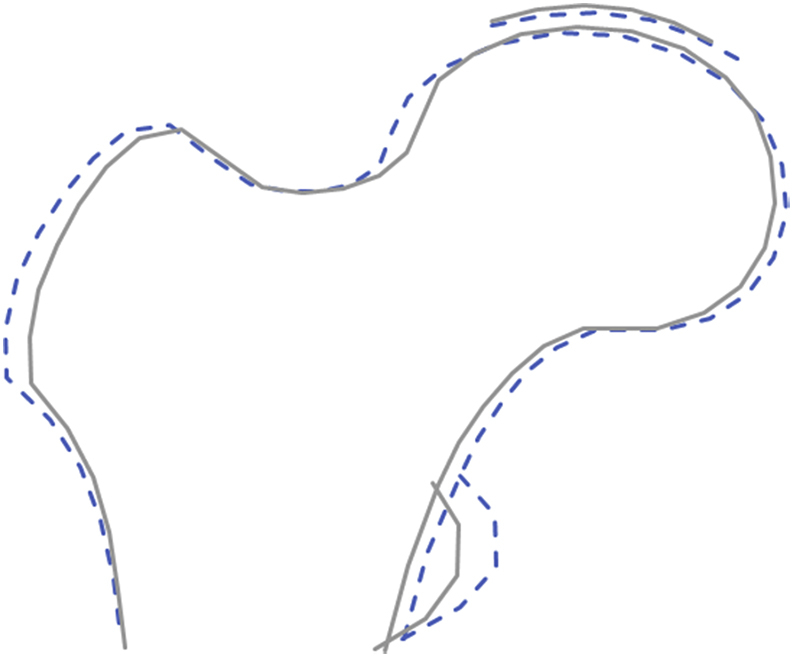
OA risk shape, The dashed line represents a composite shape formed of all the hip shape modes that were associated with rHOA in MrOS. Four modes went into this composite shape (HSM 2,3,4 & 9) with their betas multiplied by 5 for illustrative effect. The solid line represents the SSM mean hip shape for MrOS participants^21^.

HSM2 (OR 0.81, 95% CI [0.73.0.91]), HSM3 (OR 0.79, 95% CI [0.70.0.89]) and HSM4 (OR 0.72, 95% CI [0.64.0.80]) were all negatively associated with rHOA in unadjusted analyses (Table II). Following adjustment for investigator site, age, height, weight and race, HSM9 was added, which showed a positive relationship with rHOA (OR 1.20, 95% CI [1.07–1.35]) (Table II). A composite shape model visualising the combined adjusted associations suggests rHOA is related to a cam morphology, characterised by protuberance of the lateral aspect of the femoral head, and a larger lesser trochanter (Fig. 2).

**Table II:** The table shows results of logistic regression analysis between 2D hip shape modes (HSMs) and Croft score ≥2 in 4,098 individuals. Results show odds ratio of having a Croft score ≥2 per standard deviation increase in hip shape mode [95% confidence intervals] and *P-*value. Adjusted = adjusted analysis for age, weight, height, site and race. ∗*P* < 0.005

	Unadjusted Croft ≥2		Adjusted Croft ≥2	
	OR [95% CI]	*P*	OR [95% CI]	*P*
HSM 1	1.08 [0.86–1.36]	0.52	1.11 [0.87–1.40]	0.41
HSM 2	0.81 [0.73–0.91]	2.05 × 10^−04^∗	0.82 [0.74–0.92]	8.04 × 10^−04^∗
HSM 3	0.79 [0.70–0.89]	1.28 × 10^−04^∗	0.76 [0.67–0.86]	1.51 × 10^−05^∗
HSM 4	0.72 [0.64–0.80]	1.24 × 10^−08^∗	0.71 [0.63–0.79]	8.83 × 10^−09^∗
HSM 5	0.95 [0.84–1.08]	0.47	1.03 [0.91–1.17]	0.64
HSM 6	1.12 [0.98–1.28]	0.10	1.06 [0.93–1.22]	0.38
HSM 7	0.94 [0.82–1.07]	0.34	0.92 [0.81–1.06]	0.25
HSM 8	0.92 [0.82–1.04]	0.18	0.95 [0.84–1.07]	0.39
HSM 9	1.13 [1.01–1.26]	0.03	1.20 [1.07–1.35]	2.08 × 10^−03^∗
HSM 10	1.00 [0.88–1.13]	0.98	0.95 [0.84–1.09]	0.48

### Relationship between subregional hip shape modes and radiographic osteoarthritis

**Fig. 3:**
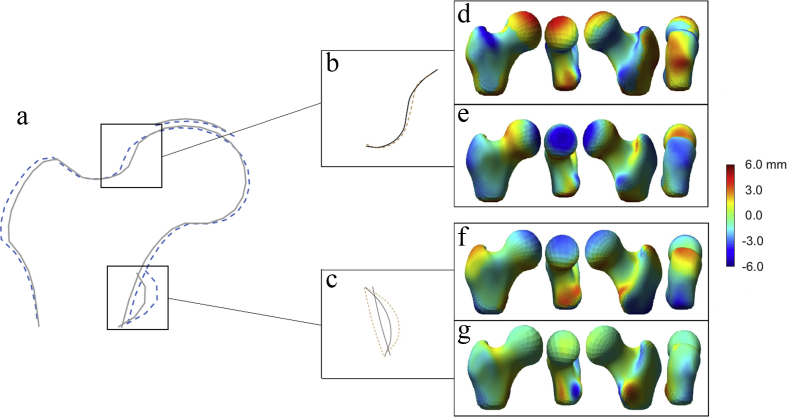
Subregional models and their associated 3D hip shape modes, a) Composite at-risk-shape for radiographic hip OA with boxes highlighting the two areas that formed our subregional SSM. b) CTM 3 pictured. The solid line represents +2 SD and the dashed line represents −2 SD. c) LTM 1 pictured. The solid line represents +1 SD and the dashed line represents −1 SD. d), e), f) & g) are 3D-HSM3, 6, 7 and 9 respectively. The heat map represents the change in shape for 1 SD movement away from the mean. The colour bar ranges from −6 to 6 mm with the average 3D femur as the base. If the point wise difference is negative (colour is towards the blue end of the spectrum) then the position of the femur surface for 1 standard deviation of the 3D-HSM is located inside the surface corresponding to mean 3D shape at that point (i.e., smaller than the mean femur at this site). If the colour tends towards red, then the femur surface for 1 standard deviation of the 3D-HSM is located outside the surface corresponding to mean 3D shape at that point (i.e., larger than the average femur at this site).

In unadjusted analyses, LTM1 (inversely related to lesser trochanter size) was negatively associated with rHOA (OR 0.74, 95% CI [0.63.0.87], *P* = 2.26 × 10^−04^) (Table III), suggesting a larger lesser trochanter is associated with a greater prevalence of rHOA [Fig. 3(c)]. Similar results were observed after adjustment for age, weight, height, site and race. No other LTMs showed association with rHOA.

**Table III:** The table shows results of logistic regression analysis between 2D subregional models (Lesser trochanter modes 1–5 & cam-type modes 1–5) and Croft score ≥2 in 4,098 individuals. Results show odds ratio of having a Croft score ≥2 per standard deviation increase in hip shape mode [95% confidence intervals] and *P-*value. Adjusted = adjusted analysis for age, weight, height, site and race. ∗*P* < 0.005

	Unadjusted Croft ≥2		Adjusted Croft ≥2	
	OR [95% CI]	*P*	OR [95% CI]	*P*
Lesser trochanter modes				
LTM 1	0.74 [0.63–0.87]	2.26 × 10 ^−04^∗	0.78 [0.67–0.92]	3.45 × 10 ^−03^∗
LTM 2	1.04 [0.89–0.78]	0.64	1.02 [0.87–1.21]	0.78
LTM 3	1.08 [0.93–0.40]	0.32	1.07 [0.92–1.25]	0.40
LTM 4	1.04 [0.94–0.51]	0.49	1.04 [0.93–1.16]	0.51
LTM 5	1.00 [0.88–0.59]	0.95	1.04 [0.91–1.18]	0.59
Cam-type modes				
CTM 1	1.19 [1.04–1.37]	0.01	1.21 [1.05–1.39]	0.01
CTM 2	0.91 [0.81–1.02]	0.09	0.90 [0.80–1.02]	0.09
CTM 3	1.27 [1.13–1.42]	7.89 × 10 ^−05^∗	1.25 [1.11–1.40]	2.24 × 10 ^−04^∗
CTM 4	1.01 [0.90–1.14]	0.83	1.03 [0.91–1.16]	0.61
CTM 5	1.11 [0.96–1.28]	0.17	1.11 [0.96–1.28]	0.17

Similarly, in unadjusted analyses, CTM3 (positively related to cam morphology) was positively associated with rHOA (OR 1.27, 95% CI [1.13.1.42], *P* = 7.89 × 10^−05^), suggesting cam morphology of the femoral head is associated with a greater prevalence of rHOA [Fig. 3(b)]. Similar results were seen in adjusted analyses. No other CTMs showed association with rHOA.

### Relationship between 2D subregional models and 3D models of hip shape

LTM1 was inversely associated with 3D-HSM7 (beta (*β*) = −0.23, 95% CI [-0.33,-0.14], *P* = 3.1 × 10^−6^) which was positively related to lesser trochanter size [Fig. 3(f)]; and positively associated with 3D-HSM9 (*β* = 0.36, 95% CI [0.27.0.45], *P* = 3.0 × 10^−13^) which was inversely related to lesser trochanter size [Fig. 3(g)]. Hence, greater lesser trochanter size from 2D DXA images, reflected by LTM1, was related to greater lesser trochanter size from 3D CT images, reflected by 3D-HSM7 and 9.

CTM3 was inversely associated with 3D-HSM3 (*β* = −0.16, 95% CI [-0.25,-0.07], *P* = 2.9 × 10^−4^) which was inversely related to cam morphology [Fig. 3(d)]; and positively associated with 3D-HSM6 (*β* = 0.19, 95% CI [0.10.0.28], *P* = 2.0 × 10^−5^) which was positively related to cam morphology [Fig. 3(e)]. Hence, cam morphology of the femoral head from 2D DXA images, reflected by CTM3, was related to equivalent appearances on 3D CT images, reflected by 3D-HSM3 and 6. No other 3D-HSMs were associated with either of these 2D subregional modes at our Bonferroni adjusted *P*-value threshold of *P* < 0.00125.

## Discussion

In a large cross-sectional study of men, we derived a comprehensive 2D at-risk-shape for prevalent rHOA by building a composite shape of those HSMs associated with rHOA. This at-risk-shape for prevalent rHOA highlighted the lateral femoral head and lesser trochanter as anatomical areas of interest. We then modelled shape variation in these areas using subregional SSM which confirmed a larger lesser trochanter and cam morphology were associated with rHOA as strongly as variation derived from whole hip shape models. These findings suggest that anatomical variation of the lateral femoral head and lesser trochanter underlie previously reported relationships between overall hip shape and prevalent rHOA.

Cam morphology, comprising a protuberance of the lateral femoral head to form a so called pistol-grip appearance has consistently been linked with rHOA, whether defined by traditional alpha angles on radiographs or from SSM^7^^,^^13^^,^^39^^,^^40^. Our findings appear to replicate these, but whether pincer morphology, an additional component of FAI syndrome, is similarly associated is unclear^41^. Our composite at-risk-shape showed no clear variation in acetabular coverage, suggesting neither pincer morphology nor acetabular dysplasia (i.e., under-coverage of the femoral head by the acetabulum) are key components of hip shapes associated with rHOA. This is contrary to the conclusions previously drawn from our SSM study in MrOS^9^ and those looking at geometric measures of these features (^42^). However, combining HSMs into a composite shape might be a poor method for exploring these deformities as both extremes of acetabular coverage are considered risk factors for hip OA, and it could be that their effects cancel each other out.

Several earlier studies examining relationships between hip shape, assessed by whole hip SSMs, and hip OA have shown HSMs with larger lesser trochanters are associated with OA^9^^,^^14^^,^^43^. However, these OA-associated HSMs also reflected other shape changes which were assumed to play a more prominent role in driving the association. A possible relationship between lesser trochanter size and risk of hip OA may have been ignored due to the assumption that lesser trochanter size is largely artefactual in that it might reflect impaired internal rotation (incomplete internal hip rotation during a DXA scan results in the appearance of a larger lesser trochanter on the image). Therefore, in order to address limitations in using 2D projections obtained from DXA scans to describe hip shape, we sought to identify 3D modes of variation in hip shape with which these are associated, derived from hip CT scans performed in the same individuals^18^. Interestingly, in the case of both our sub-regional models, the 3D-HSMs with which they had the strongest evidence of association showed the equivalent direction of variation in lesser trochanter size/cam morphology. These exploratory findings, from a cross-sectional analysis, raise the possibility that lesser trochanter size on DXA could represent a true risk factor for rHOA, as opposed to an artefact resulting from incomplete internal rotation. Although the mechanisms responsible for such a relationship are currently unclear, this may point to a previously unrecognised contribution of the iliopsoas muscle. Previous cadaveric studies have shown the psoas major muscle, which inserts into the lesser trochanter, not only flexes the hip but stabilises it by controlling the pressure through the acetabulum and femoral head^44^. Taken with our findings, over time, aberrant biomechanical forces mediated through the lesser trochanter might contribute to the development of rHOA.

### Strengths and limitations

Strengths of this study include the large sample size and our application of novel SSMs for subregional hip shape, which concentrate on one distinct area of anatomy, and enable a more precise description of shape variation associated with OA. This increases our understanding of the pathogenic mechanisms of hip shape variation which may in turn inform clinical interventions focused on hip shape^45^^,^^46^. The use of a 5-cohort reference SSM^21^ means this study could be replicated in other cohorts, unlike SSMs derived from a single cohort, however our results cannot be directly compared to a previous publication that used an SSM solely based on MrOS^9^. Another strength is the validation of our DXA-derived 2D-HSMs against 3D-HSMs derived from hip CT scans. In terms of weaknesses, as this study was conducted only in males, we cannot infer that equivalent relationships between localised hip shape and rHOA exist in females. Although we adjusted for known confounders, in common with other observational studies we are unable to account for unknown or unmeasured confounders. Multiplicity is an inherent problem with SSM as many HSMs are generated, we had a strategy to reduce any type I error and feel this adds strength to our conclusions though of course the possibility remains we have not fully eliminated its effects.

The absence of baseline radiographs meant we were only able to examine associations with prevalent, as opposed to incident, OA. Hence, the hip shape differences we observed could be a consequence rather than a cause of rHOA. To explore possible causal relationships between hip shape and rHOA, future studies might employ Mendelian Randomisation (MR), a technique that uses genetic variants as proxies for risk factors of disease which would mitigate the effect of unmeasured confounding^47^. A recent genome-wide association study of hip shape measured by SSM found 8 independent genetic loci that show such genetic proxies for SSM-derived hip shape exist^21^. To the extent that genetic proxies can also be identified for sub-regional hip shape, it may be possible to apply MR to explore whether associations between lesser trochanter size and cam-shape femoral head and rHOA, which we found, represent a causal role of these anatomical features in the development of hip OA.

In terms of other weaknesses, when looking at 2D subregional associations with 3D proximal femur shape we cannot be sure the same anatomical areas are driving this association, and further work is needed to test this assumption. By limiting our analysis to the first 5, 10 or 20 modes we do not test all of the shape variance captured by each SSM, but this is justified to limit multiple testing and focus our attention on larger anatomical variations. Determining associations between 3D-HSMs and rHOA would strengthen our conclusions, however there were only 28 cases of rHOA in the current study sample and so we were underpowered to look for these associations. In addition, whereas the present study focused on associations with rHOA, radiographic changes of OA are not that strongly related to clinical consequences such as pain and stiffness^48^.

## Conclusions

Analysis of a composite hip shape, obtained from SSM, applied to hip DXA scans in a large cross-sectional study of older men, suggested that larger lesser trochanter size and cam morphology of the femoral head are both associated with higher prevalence of rHOA. This conclusion was supported by findings from subregional models that were subsequently built describing both such deformities and which showed similar strength associations with rHOA as we saw with whole HSMs. Further studies are justified to examine lesser trochanter size as a potentially novel determinant of rHOA, including the possible role of altered biomechanics.

## Contributions

All authors have made significant contributions to the conception and design of this study, the acquisition of data, its analysis and interpretation. All authors helped draft the article before approving the final version of this manuscript. Dr B Faber (ben.faber@bristol.ac.uk) takes responsibility for the integrity of the work in its entirety.

## Conflict of interest

There are no conflicts of interest to declare.
