# Supplementary material for: Subregional statistical shape modelling identifies lesser trochanter size as a possible risk factor for radiographic hip osteoarthritis, a cross-sectional analysis from the Osteoporotic Fractures in Men Study
Source: Osteoarthritis Cartilage. 2020 Aug;28(8):1071–8. doi: 10.1016/j.joca.2020.04.011 (PMC7387228; doi:10.1016/j.joca.2020.04.011)
Supplement: Supplementary file 1 — Multimedia component 1 [file mmc1.docx]

| 3D-HSM | *r^2^* |
| --- | --- |
| 1 | 0.10 |
| 2 | 0.12 |
| 3 | 0.15 |
| 4 | 0.10 |
| 5 | 0.15 |
| 6 | 0.26 |
| 7 | 0.63 |
| 8 | 0.05 |
| 9 | 0.17 |
| 10 | 0.07 |
| 11 | 0.12 |
| 12 | 0.02 |
| 13 | 0.04 |
| 14 | 0.09 |
| 15 | 0.09 |
| 16 | 0.03 |
| 17 | 0.05 |
| 18 | 0.06 |
| 19 | 0.06 |
| 20 | 0.05 |

**Supplementary Table 1.**

The table shows variance explained, represented by r-squared (r^2^), of each 3-dimensional hip shape mode (3D-HSM) by our 2-dimensional (2D) whole hip shape model. This was calculated using multiple linear regressions with 2D hip shape modes 1-10 as our exposure and each 3D-HSM as our outcome.

**Supplementary Figure 1**: Lesser trochanter modes


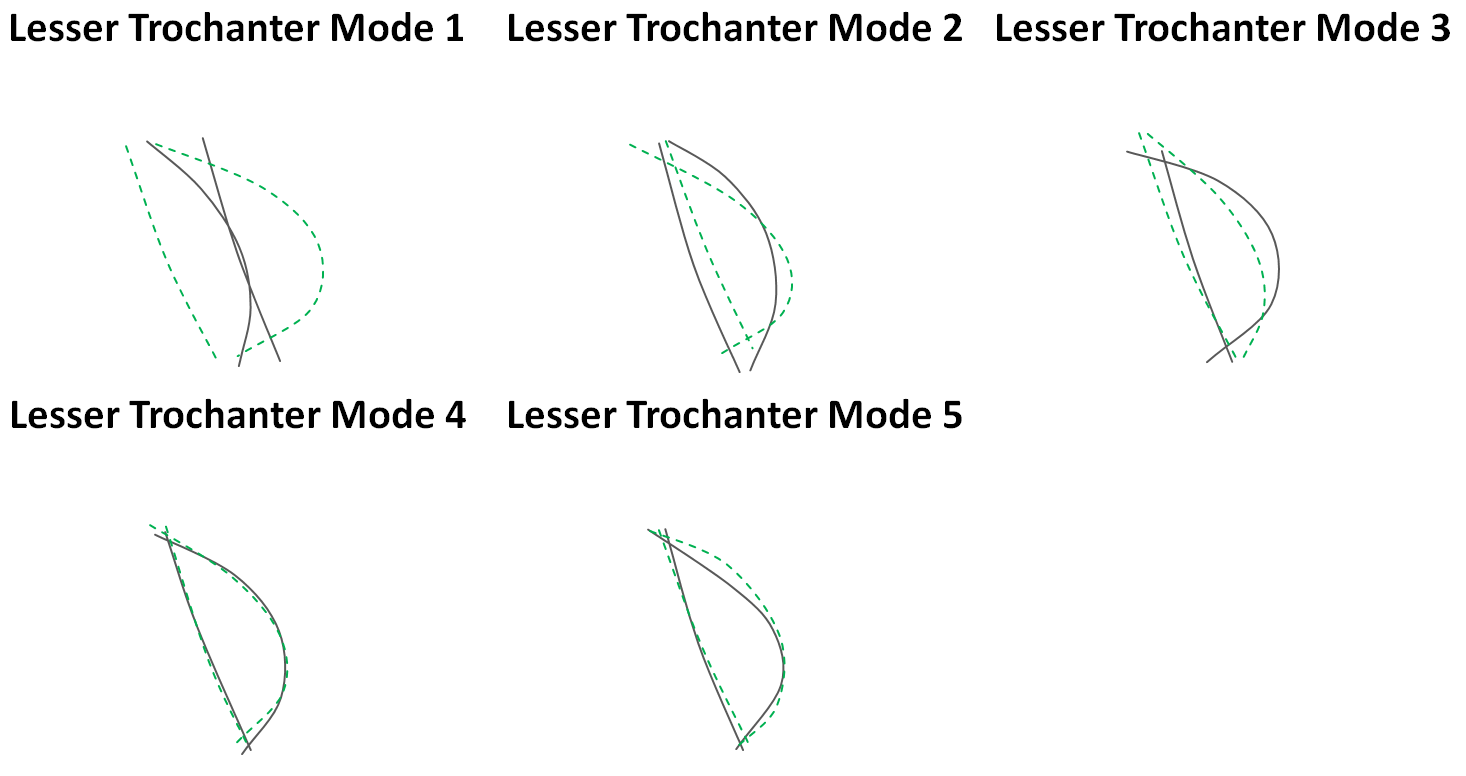


The figure represents all five lesser trochanter modes analysed. The solid line represents +2 SD and the dashed line represents -2 SD.

**Supplementary Figure 2**: Cam-type modes


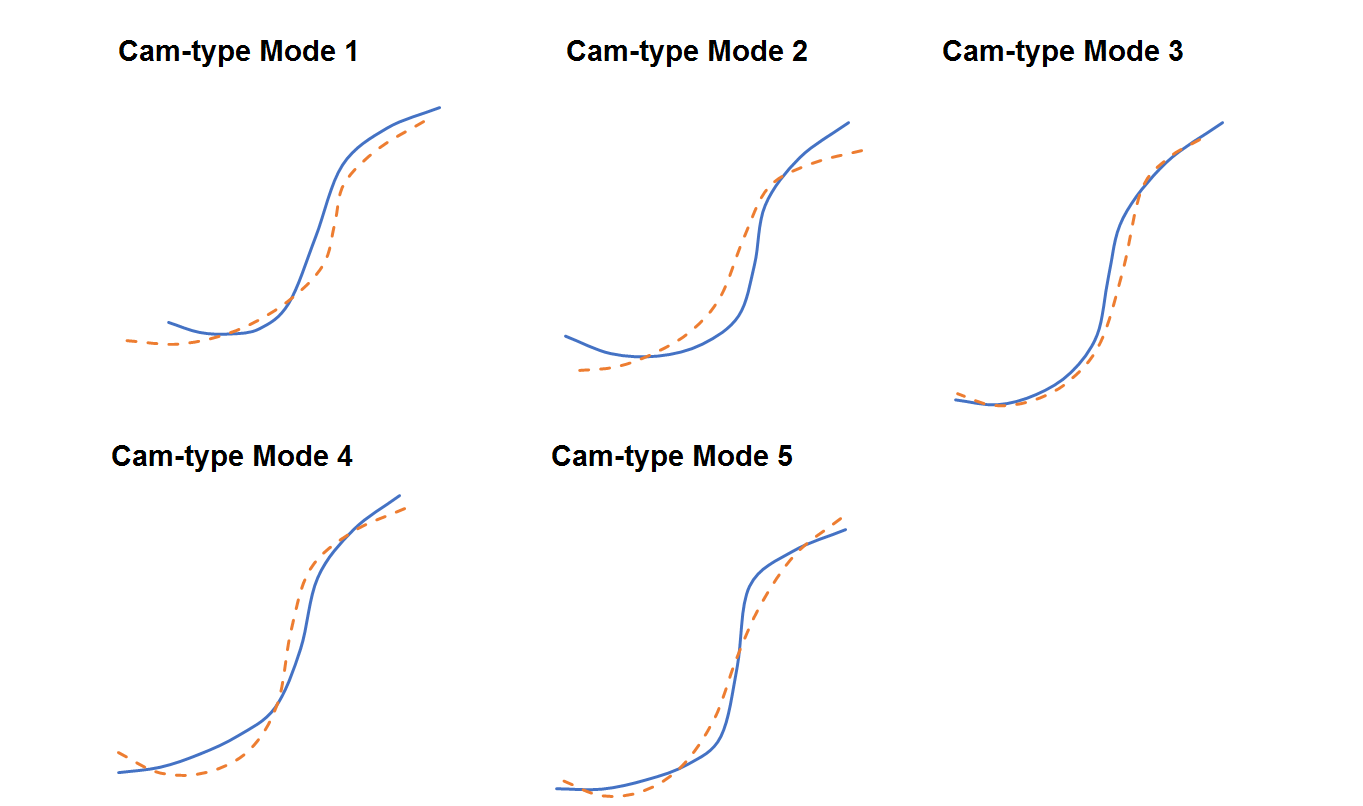


The figure represents all five cam-type modes analysed. The solid line represents +2 SD and the dashed line represents -2 SD.

**Supplementary Figure 3**: 3D Hip Shape Modes


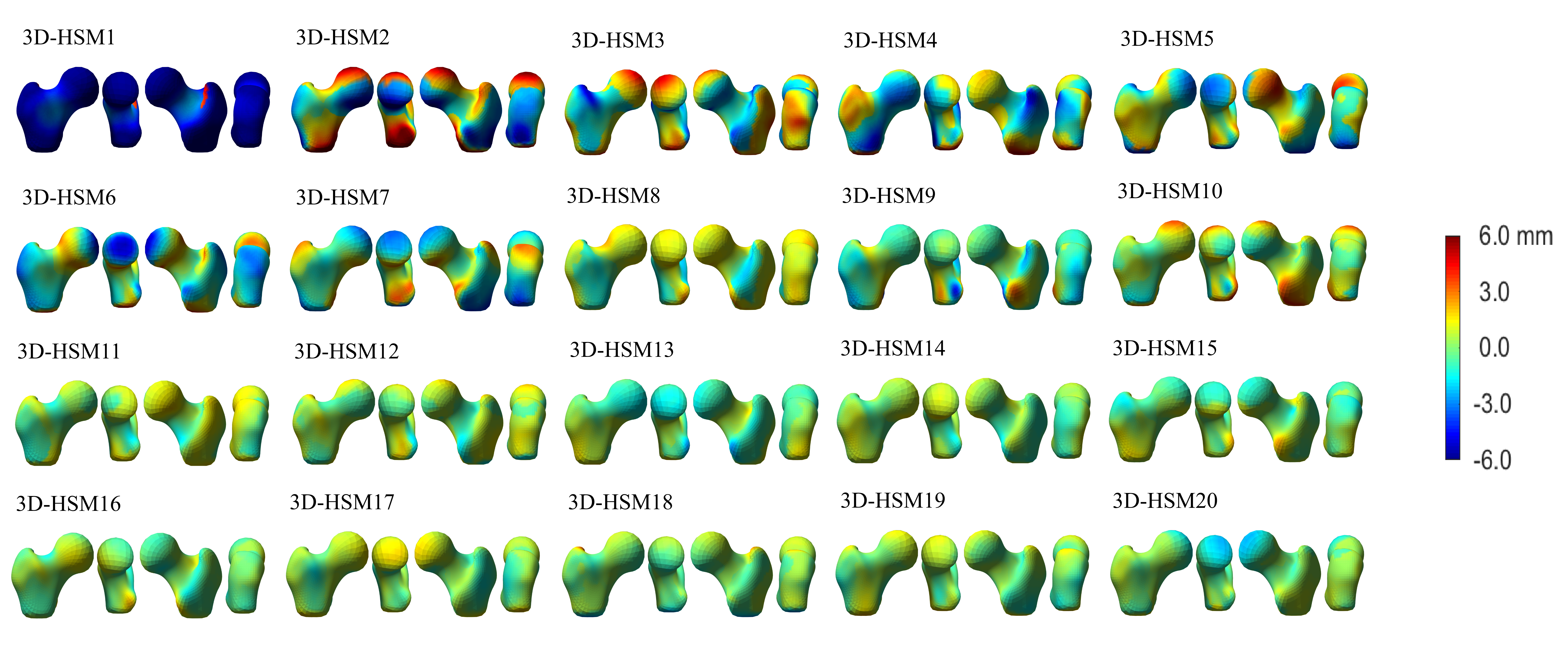


The figure represents all twenty 3-dimensional hip shape modes (3D-HSMs) analysed.


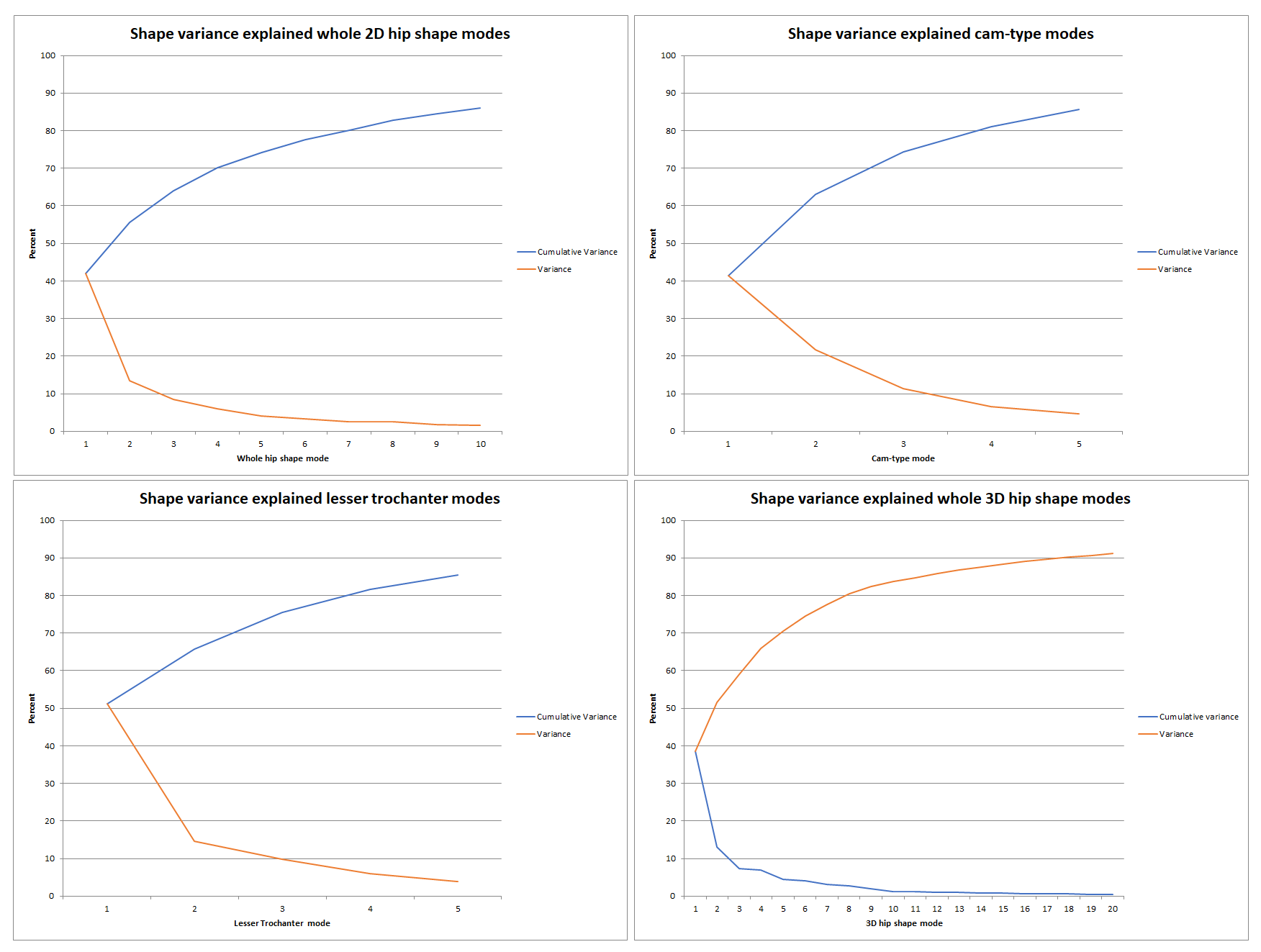
**Supplementary Figure 4**: Scree plots

The figure represents the total shape variation explained by each statistical shape model including the amount of variation explained by each mode examined.
